# Treatment of engineering waste slurries by microbially induced struvite precipitation mechanisms

**DOI:** 10.3389/fbioe.2023.1109265

**Published:** 2023-01-20

**Authors:** Yuhan He, Shiyu Liu, Gangqiang Shen, Muzhi Pan, Yanyan Cai, Jin Yu

**Affiliations:** ^1^ College of Civil Engineering, Huaqiao University, Xiamen, China; ^2^ Fujian Water Conservancy and Hydropower Engineering Bureau Company Limited, Quanzhou, China

**Keywords:** MISP, flocculation, waste mud, *Sporosarcina pasteurii*, struvite

## Abstract

With societal development, the growing scale of engineering construction, and the increase in environmental protection requirements, the necessity of engineering waste mud disposal is becoming increasingly prominent. In this study, microbially induced struvite precipitation (MISP) was introduced to treat engineering waste mud. The study mainly focused on: i) the optimal mineralization scheme for microbially induced struvite precipitation, ii) the feasibility of the process and the effect of reaction parameters on treating engineering waste mud with microbially induced struvite precipitation, and iii) the mechanism of microbially induced struvite precipitation in treating engineering waste mud. The results showed that the waste mud could be well treated with $8.36\times 10^{6}\;cell\cdot {mL}^{-1}$ bacteria, 10 mM urea, 20 mM phosphate buffer, and 25 mM ${MgCl}_{2}$ at pH 7. The kaolin suspension could be effectively flocculated. The flocculation rate reached approximately 87.2% under the optimum mineralization conditions. The flocculation effect was mainly affected by the concentrations of reactants and heavy metals and the suspension pH. The X-ray diffraction (XRD) patterns showed a strong struvite (MAP) diffraction peak. Scanning electron microscopy (SEM) images indicated that under the optimal mineralization conditions, the crystals were large and showed prismatic shapes tilted at both ends with adhered kaolin particles. In summary, this manuscript provides an effective way to treat engineering waste mud, and the findings should have a positive effect on enhancing soil fertility and preventing secondary pollution.

## 1 Introduction

**FIGURE 1:**
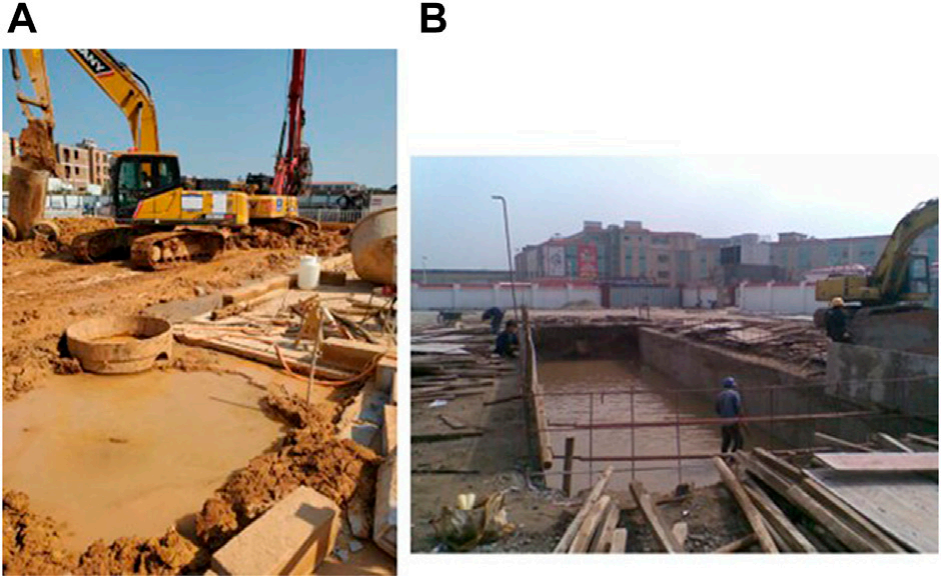
**(A)** Waste mud that has been dug up and **(B)** mud pool.

With increasing societal development, the scale of engineering construction is continuing to expand, resulting in the production of greater amounts of construction mud (e.g., shield soil). According to a previous study, 300 million $m^{3}$ of construction mud is produced in China (Rui et al., 2020). Therefore, without the proper disposal of waste mud, the surrounding environment will be polluted to a large degree. As shown in Figure 1A, the clay and moisture contents of the engineering waste mud were high. The dehydration of the waste mud was difficult, and the particles of the waste mud were anisotropic (Ding et al., 2022). The mud produced on site was usually transferred to the mud pool (Figure 1B).

Flocculation is an important process in treating engineering waste mud. To date, there are various flocculants, including inorganic flocculants, organic flocculants, and microbial flocculants (Lee et al., 2014; Dotto et al., 2019). Inorganic flocculants, such as aluminium and ferric polymers, are economically friendly and easy to obtain, but they have negative effects on water quality. Organic flocculants essentially refer to organic polymer flocculants, and polyacrylamide and its derivatives are the primary organic polymer flocculants. Specifically, polyacrylamide and its derivatives can be split into non-ionic, cationic, anionic, and amphoteric types. They can significantly accelerate the flocculation process at a low dosage and are convenient to separate from water (Wei et al., 2018). Although possessing a better pH in application than inorganic flocculants (Faouzi et al., 2018), organic polymer flocculants are difficult to degrade and could further cause secondary environmental pollution. Previous studies had found that chemical methods using calcium and sodium salts had adverse effects on the microstructure evolution and macroscopic mechanical properties of silt soils (Wang et al., 2022a).

Microbial flocculants are safe, non-toxic and environmentally friendly and are considered ideal substitutes for inorganic and organic polymer coagulants (Zhao et al., 2017). Therefore, as a product with centuries of history, microbial flocculants may have extensive application potential in treating engineering waste mud. Generally, non-toxic biopolymers formed by microorganisms or their metabolites are the active components of microbial flocculants. The flocculation efficiency is positively correlated with the length of the biopolymer flocculant molecular chain; that is, the longer the molecular chain is, the better the flocculation efficiency (Li et al., 2020). With advances in science and technology, various microbial flocculants have been investigated and developed. Traditional microbial flocculants are metabolites produced by microorganisms and do not participate directly in the flocculation process. Thus, it is necessary to strip the active ingredient from the microbial medium to explore the mechanisms of flocculation. For example, flocculants can be separated and purified from microbial media through organic solvent precipitation and the Sevage method (Wei, 2017). However, these methods are complicated and difficult to perform.

Phosphate (P) and ammonia (N) are often present in actual water bodies and cause eutrophication at high concentrations (Mavhungu et al., 2021). Therefore, the recovery of P and N is an important process in water treatment. In this study, microbially induced struvite precipitation (MISP) was introduced to treat engineering waste mud. In contrast to traditional microbial flocculants, MISP technology can realize the direct participation of bacteria in the flocculation process. The MISP technique is one of the biomineralization techniques, along with microbially induced calcite precipitation (MICP) and enzyme-induced carbonate precipitation (EICP). Previous studies had explored reinforcing rocks and soils with MICP technology (Tang et al., 2020; Xiao et al., 2021). EICP technology was used to increase soil strength, and the addition of Mg^2+^ could enhance the improvement effect (Hu et al., 2021). In addition, Electrokinetic treatment (EKT) and Biopile treatment, excellent soil remediation techniques, effectively degraded heavy metals and organic matter in the soil (Gandhi et al., 2022; Mohammad et al., 2022). In previous studies, straw fiber could be used to increase the strength of soil (Xue et al., 2021).

Microorganisms, equipped with negative charges, can transform nitrogen-containing compounds to ${NH}_{4}^{+}$ through metabolic activities and then adsorb cations from the liquid phase. The cations combine with phosphate ions in solution to form struvite (MAP) with certain cohesion properties (Yu et al., 2016). Specifically, MAP can be used as a precipitation agent in water bodies to eliminate eutrophication (Luo et al., 2018). MISP mainly has two mechanisms. First, microorganisms with negative charges on their surfaces serve as nucleation sites of MAP precipitation and attract cations from the solution. Second, urease is generated during the process of microorganic metabolism and catalyzes the hydrolysis of nitrogen-containing compounds to ${NH}_{4}^{+}$ *via* Eqs 1–3, which is conducive to the production of MAP ( ${MgNH}_{4}{PO}_{4}\cdot 6H_{2}O$ ) (Eq. 4) (Yu et al., 2021; Wang et al., 2022b). A previous study reported that MAP could be precipitated by adding NaOH to adjust the pH of the system (Yao, 2006), while the generated alkaline MAP could seriously damage the soil and, thus, limit its potential as a compound fertilizer for agriculture (Leng and Soares, 2021). Surprisingly, MISP can produce a solid phase that can serve as a fertilizer containing abundant nitrogen and phosphorus and further minimize environmental pollution. Moreover, MISP technology can reduce ammonia pollution (Dong et al., 2020) and enhance flocculation efficiency.

$${NH}_{2}{CONH}_{2}+H_{2}O\overset{Ureolytic\;bacteria}{\to }{NH}_{3}+{NH}_{2}COOH$$ (1)

$${NH}_{2}COOH={NH}_{3}+{CO}_{2}$$ (2)

$${NH}_{3}+H_{2}O⇌{NH}_{4}^{+}+{OH}^{-}$$ (3)

$${Mg}^{2+}+{PO}_{4}^{3-}+{NH}_{4}^{+}+6H_{2}O⇌{MgNH}_{4}{PO}_{4}\cdot 6H_{2}O$$ (4)

This study introduced MISP to treat engineering waste mud and mainly focused on: i) the optimal mineralization scheme for MISP, ii) the feasibility and the effect of reaction parameters on treating engineering waste mud with MISP, and iii) the mechanism of MISP in treating engineering waste mud. The microbial and MISP mechanisms were studied by X-ray diffraction (XRD), scanning electron microscopy (SEM), and other characterization methods.

## 2 Materials and methods

### 2.1 Materials

All initial inorganic reagents were of analytical grade and purchased from Sinopharm Chemical Reagent Co., Ltd. The yeast extract was of biotechnology grade and was purchased from Sangon Biotech (Shanghai) Co., Ltd. All chemicals were used as purchased, without further purification. Bacteria were purchased from the German Biological Collection. Deionized water was used in all experiments.

### 2.2 Bacterial strain, culture medium, and cultivation

*Sporosarcina pasteurii* is a facultative anaerobic Gram-positive bacterium with a high urease secretion capacity (Yang, 2013), good adaptability in the environment, and high urease activity and mineralization efficiency, which is often used to reinforce rocks and soils *via* MICP (Mountassir et al., 2018; Liu and Gao, 2020). This bacterium was selected for the experiments in this study.

ATCC 1376 NH_4_-YE medium (Wang et al., 2019) was used to culture *S. pasteurii.* For the study, 100 mL of the culture medium was placed in a 250 mL conical flask and autoclaved at 121°C for 40 min. Then, the strain was inoculated into the culture medium, covered with breathable film, and cultured at 30°C and 180 rpm for 48 h to prepare a stable liquid as the seed liquid for subsequent experiments.

In this study, the concentration of bacteria was calculated by using the optical density of the bacterial solution at 600 nm (OD_600_) (Zhao, 2014). When OD_600_ = 1.5, the number of microorganisms was considered to be stable (Sarda et al., 2009).

### 2.3 Experimental procedures

To explore the optimal mineralization parameters, feasibility, and mechanisms of MISP during flocculation, the concentration of the added bacterial solution was first determined. Then, we explored the optimal scheme and feasibility for the treatment of engineering waste mud with MISP, as well as the influence of pH and heavy metal ions on the treatment efficiency. Finally, the mechanisms were analyzed through microscopic characterization.

#### 2.3.1 Determination of bacterial concentration

To obtain the optimal bacterial concentration conducive to mineralization, mineralization tests were designed with different bacterial concentrations using a direct ammonium source ( ${NH}_{4}Cl$ ) and an indirect ammonium source (urea). The test conditions are shown in Table 1. The solution pH was controlled with phosphate buffer (PBS; $K_{2}{HPO}_{4}$ / ${KH}_{2}{PO}_{4}$ ) at pH 7, and then magnesium chloride was added. Additionally, both PBS and magnesium chloride served as the reactants for MAP precipitation.

**TABLE 1:** Different concentrations of microorganisms and reactants added to each sample.

Group	Sample	Bacterial liquid (mL)	OD_600_	Culture medium	Bacteria (cell·ml^−1^)	NH_4_Cl (mM)	Urea (mM)	PBS (pH 7, mM)	MgCl_2_ (mM)
A	A0	3	0.096	-	3.49×10^6^	2	0	4	5
	A1		0.096	-	3.49×10^6^	2	0	4	0
	A2		-	3%	0	2	0	4	5
	A3		0.096	-	3.49×10^6^	0	2	4	5
B	B0	5	0.181	-	8.30×10^6^	2	0	4	5
	B1		0.181	-	8.30×10^6^	2	0	4	0
	B2		-	5%	0	2	0	4	5
	B3		0.181	-	8.30×10^6^	0	2	4	5
C	C0	10	0.300	-	1.66×10^7^	2	0	4	5
	C1		0.300	-	1.66×10^7^	2	0	4	0
	C2		-	10%	0	2	0	4	5
	C3		0.300	-	1.66×10^7^	0	2	4	5

${NH}_{4}^{+}$ was added to the system as the direct ammonium source. The specific steps were as follows: 3 mL, 5 mL, and 10 mL of stabilized bacterial solution were added to 97 mL, 95 mL, and 90 mL of a mixed solution composed of ${NH}_{4}Cl$ , PBS (pH 7), and ${MgCl}_{2}$ , respectively (Dong et al., 2020). The initial concentrations of ${NH}_{4}Cl$ , PBS (pH 7), and ${MgCl}_{2}$ were 2 mM, 4 mM, and 5 mM, respectively, in A0, B0, and C0, as noted in Table 1. The solutions were reacted at 30°C for 3 h. In addition, the Phospholipid bilayer is the main structure of a bacterial cell membrane, while the P element is the main component of the Phospholipid bilayer. Therefore, it is necessary to explore the cell’s consumption of phosphorus. The initial concentrations of ${NH}_{4}Cl$ , PBS (pH 7), and ${MgCl}_{2}$ were 2 mM, 4 mM, and 0 mM, respectively, in A1, B1, and C1, as noted in Table 1.

Due to their ability to convert urea in the environment to ${NH}_{4}^{+}$ , the metabolic activities of urease-producing bacteria were used to provide indirect ammonium sources (Liu and Gao, 2020). Urea is widely found in aquaculture wastewaters and domestic sewage and is a relatively inexpensive material for sustaining bacterial growth. Thus, urea was used in the MAP mineralization precipitation experiments of A3, B3, and C3. As the ${NH}_{4}^{+}$ used in MAP mineralization required bacterial urease to decompose urea, the reaction times of A3, B3, and C3 were longer than those of A0, B0, and C0. Therefore, A3, B3, and C3 were designed as noted in Table 1 and were cultured for 24 h in an incubator at 30°C and 180 rpm.

In addition, a sterile culture medium was used as the control group to exclude the influence of different volumes of culture medium on MAP mineralization. Therefore, an experiment to explore the influence of the culture medium on MAP precipitation was designed in the sterile state, and the conditions are shown in A2, B2, and C2 as noted in Table 1. The bacterial solution was filtered through a 0.22 μm acetate membrane to replace the bacterial solution that had reached a growth plateau. The other conditions were the same as noted for A1, B1, and C1 in Table 1.

The appropriate microbial concentration for MAP was determined through the above experiments for use in subsequent experiments. The apparent rate constant ( $k_{obs}$ ) is a quantitative expression of the reaction rate in chemical kinetics. The mineralization reaction was initiated by bacteria and followed *pseudo*-first-order kinetics and the corresponding $k_{obs}$ was calculated by Eq. 5 (Paris and Rogers, 1986).

$$k_{obs}=-\frac{dC}{dt}$$ (5)

where C is the concentration of the sample and *t* is the reaction time at the sample collection time point. The concentration of ${PO}_{4}^{3-}$ during the reaction was determined, and $k_{obs}$ was calculated to analyze the experimental results and determine the optimal bacterial concentration.

#### 2.3.2 Optimization of the MISP mineralization scheme

A 5 g/L kaolin suspension was used as simulated mud to optimize the scheme and analyze the effect of MISP mud treatment (Ajao et al., 2018). The experimental group was divided into six groups according to the concentration of the added reactants. The control group was divided into control group I and control group II, and bacteria were replaced with culture medium and ultrapure water, respectively. The system components of each group are shown in Table 2. Kaolin powder was added to the mixed solution composed of ${MgCl}_{2}$ , PBS, and urea, and then the suspension was agitated after the addition of the bacterial liquid obtained in Section 2.3.1 [the kaolin concentration was 5 g/L at this time (0.5% wt/vol)]. Subsequently, the conical flask containing the suspension was placed in a water bath agitator and reheated to 30°C. The concentration of ${PO}_{4}^{3-}$ , the efficiency of flocculation, and pH at different time points (0, 0.5, 1, 2, 4, 8, 12, and 24 h) were recorded.

**TABLE 2:** Conditions and concentrations of different groups.

Group	Conditions	Urea (mM)	PBS (pH 7, mM)	${MgCl}_{2}$ (mM)
Group I	Bacteria	2	4	5
Group II		4	8	10
Group III		6	12	15
Group IV		10	20	25
Group V		14	28	35
Group VI		18	36	45
Control I	Culture medium	2	4	5
Control II	Ultra-pure water			

**FIGURE 2:**
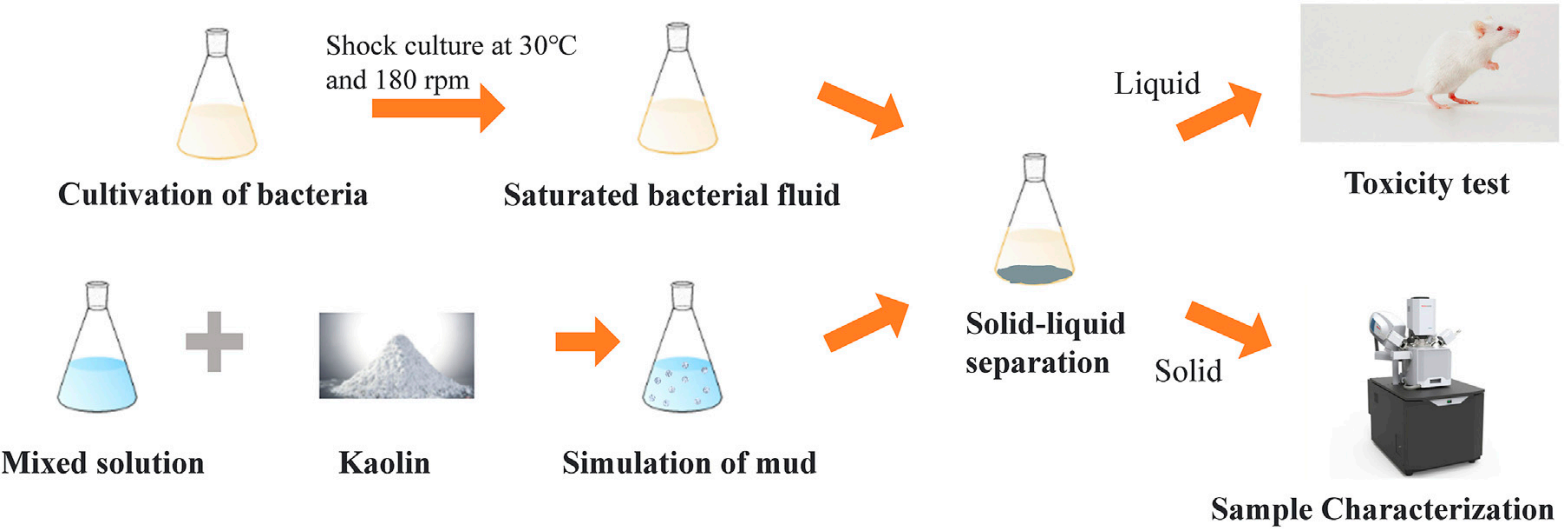
The process of waste mud disposal by MISP.

Finally, the precipitation samples obtained from the experiment were washed and dried. XRD and SEM were used to analyze the mechanisms involved in the treatment of engineering waste mud with MISP. The obtained liquid was filtered and tested in mice to analyze toxicity. The experimental method and process are shown in Figure 2.

#### 2.3.3 Effect of initial pH

The precipitation of MAP can be affected by pH. To understand the influence of pH on the flocculation effect of the treatment of engineering waste mud with MISP, the mineralization flocculation experiments under 6, 7, 8, 9, 10, and 11 pH values (adjusted by the prepared PBS ( $K_{2}{HPO}_{4}$ / ${KH}_{2}{PO}_{4}$ / $K_{3}{PO}_{4}$ )) were carried out with the best bacterial concentration and mineralization scheme described in Section 2.3.2. Then, the urea, ${MgCl}_{2}$ , ultrapure water, and kaolin were mixed to prepare the suspension. The suspension was stirred well and added the bacterial liquid. Subsequently, the conical flask containing suspension was placed in a water bath agitator and reheated to 30°C, and the concentrations of ${PO}_{4}^{3-}$ and ${NH}_{4}^{+}$ , the efficiency of flocculation, and pH at different time points (0, 0.5, 1, 2, 4, 8, 12, and 24 h) were recorded.

#### 2.3.4 Effect of $Cu^{2+}$

Heavy metal ions that enter surrounding environments can threaten human health, such as by causing kidney failure and neurological and digestive disorders in humans upon exposure (Xue et al., 2022). A previous study reported that in Hangzhou shield waste mud, heavy metal ions were prevalent, and the concentration of ${Cu}^{2+}$ was high, up to 162.14 mg/kg (Zhang et al., 2022). The activity of bacteria can be affected by heavy metal ions, so it is necessary to explore the influence of heavy metal ions on the flocculation efficiency of MISP. In this study, ${Cu}^{2+}$ was selected as the main target heavy metal, and flocculation with 0, 0.1, 0.2, 0.4, 0.6, and 0.8 mM ${CuCl}_{2}$ was assessed under the optimum bacterial solution concentration, reactant concentrations, and initial pH. Subsequently, the conical flask containing the suspension was placed in a water bath agitator and reheated to 30°C, and the concentrations of ${PO}_{4}^{3-}$ and ${NH}_{4}^{+}$ , the efficiency of flocculation, and pH at different time points (0, 0.5, 1, 2, 4, 8, 12, and 24 h) were recorded.

### 2.4 Analytical methods

#### 2.4.1 Concentrations of phosphate ( $PO_{4}^{3-}$ -P) and ammonia ( $NH_{3}$ -N)

Since the precipitation capacity in the experiment was difficult to quantitatively analyze, the ratio of the ${PO}_{4}^{3-}$ concentration at each time to that at time zero ( $C_{t}/C_{0}$ ) was used to show the change of ${PO}_{4}^{3-}$ , and the results further reviewed the mineralization capacity and MAP precipitation of the bacteria. The concentration in ${PO}_{4}^{3-}$ -P in the supernatant of the reaction system was measured by the molybdenum-antimony resistance spectrophotometric method GB 11893-1989. A 40 mL solution containing 2.6 g ammonium molybdate and 0.07 g potassium antimony tartrate was added to 60 mL diluted sulfuric acid [sulfuric acid:water (1:1)] to form a molybdate complex solution. One milliliter of ascorbic acid solution (10% wt/vol) and 1 mL of molybdate complex solution were added to 50 mL of sample solution (diluted 200 times). Subsequently, the absorbance value of ${PO}_{4}^{3-}$ -P at 700 nm was measured by a UV‒vis spectrophotometer after 10 min. The concentration of ${NH}_{3}$ -N was measured by Nessler spectrophotometry (HJ 539-2009). One milliliter of 50% potassium sodium tartrate solution and 1.5 mL of mercury-iodide Nessler reagent were added to 50 mL of sample solution (diluted 200 times). Subsequently, the absorbance value of ${NH}_{3}$ -N at 420 nm was measured by a UV‒vis spectrophotometer after 15 min.

#### 2.4.2 Flocculation rate

The flocculation rate is a dimensionless ratio and was used to denote the flocculation efficiency. At the end of the reaction, the suspension was moved to a settling tube and left undisturbed for 10 min. The absorbance value of the supernatant at 550 nm was measured by a UV‒vis spectrophotometer and used in the calculation in Eq. 6.

$$Flocculation\;rate=\frac{A_{1}-B_{1}}{A_{1}}\times 100\%$$ (6)

A_1_ is the absorbance value of the Control II supernatant at 550 nm, and B_1_ is the absorbance value of the sample supernatant at 550 nm.

#### 2.4.3 Sample analysis

Sediment was collected through a vacuum filter, washed three times with anhydrous ethanol, and dried in a drying box at 50°C. The material composition of the samples was analyzed by XRD. All XRD analyses were performed on a Miniflex 600 (Neiji Co., LTD., Japan). The scanning angle was 4°–90°, and the scanning speed was 10°/min. MDI Jade 6 Pattern Processing software was used to identify the qualitative phases. The morphology and elemental composition of the samples were studied by SEM and transmission electron microscopy (TEM). An SU 1510 SEM system (Hitachi High-Tech Co., LTD., Japan) and a Talso F200X TEM system (Thermo Fisher Scientific Co., LTD., United States) were used.

## 3 Results and discussion

### 3.1 Determination of bacterial concentration

#### 3.1.1 Direct ammonium source

**FIGURE 3:**
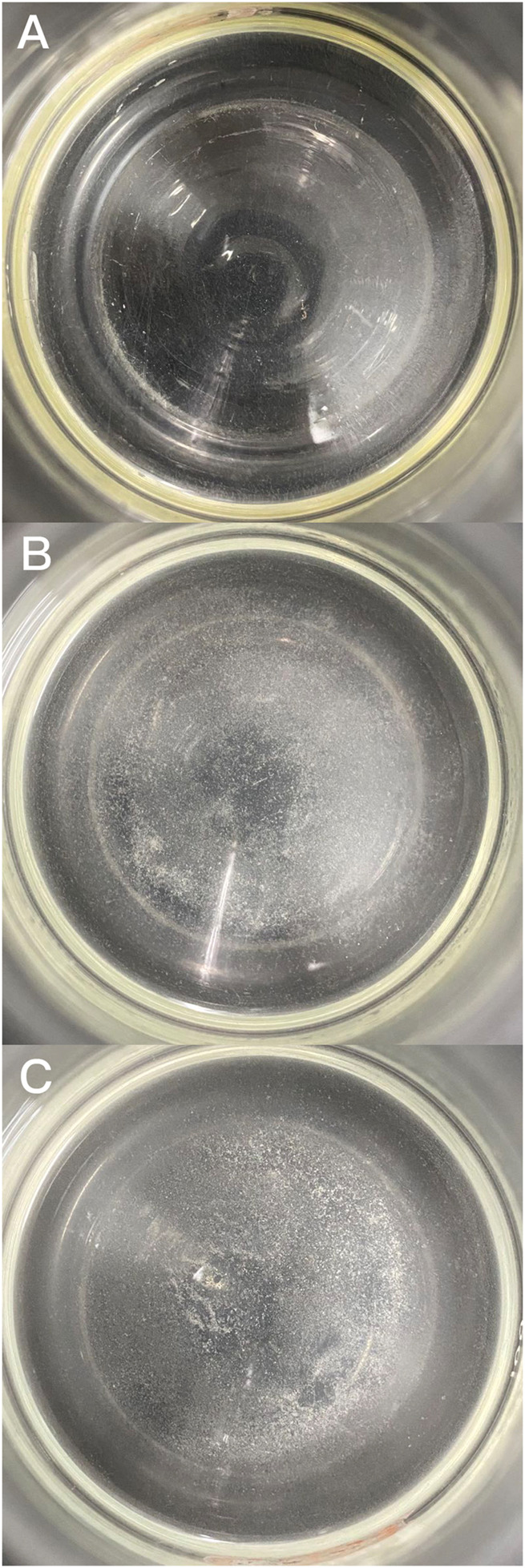
Photos of reactors **(A)** A1, **(B)** B1, and **(C)** C1.

White precipitates were observed at 20 min and significantly increased at 3 h in A0, B0, and C0. The precipitates of A0 were less than those of the other two treatments (Figure 3). This result indicated that the bacterial solution had the ability to induce precipitation in the MISP system, and the concentration of bacteria had a positive effect on the reduction in precipitation.

**FIGURE 4:**
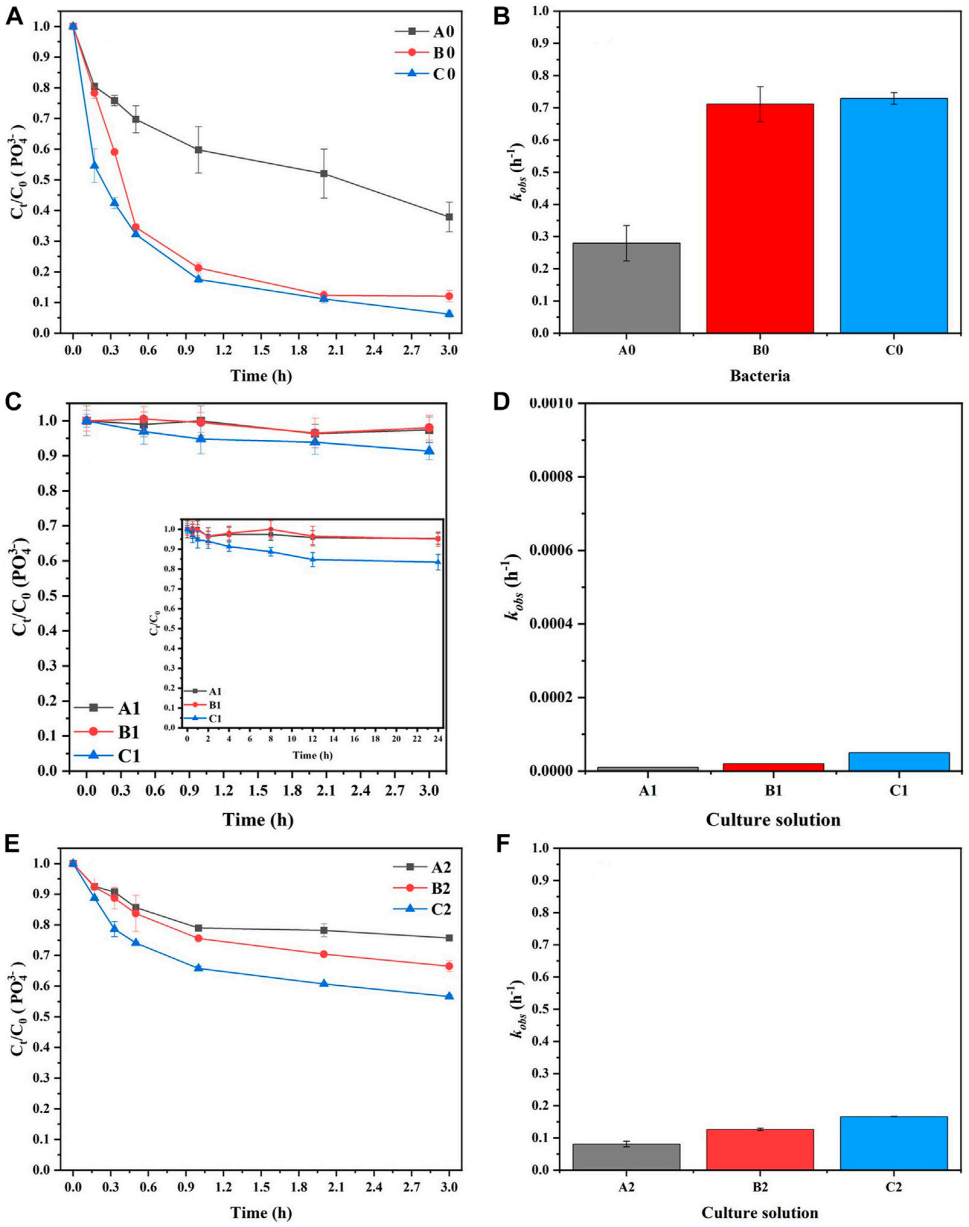
The effect of bacterial concentration on **(A)** the elimination of ${PO}_{4}^{3-}$ and **(B)** the corresponding *k*_*obs*_ value, the effect of bacteria cell membrane on **(C)** The elimination of ${PO}_{4}^{3-}$ and **(D)** the corresponding $k_{obs}$ values, and the effect of sterile culture medium concentration on **(E)** the elimination of ${PO}_{4}^{3-}$ and **(F)** the corresponding *k*_*obs*_ value with MISP.

Figures 4A, B shows the effect of various bacterial solution concentrations (A0, B0, and C0) on ${PO}_{4}^{3-}$ elimination and the corresponding apparent rate constant ( $k_{obs}$ ) values with the direct addition of an ammonium source. Due to mineralization by microorganisms, the phosphorus concentration gradually decreased, and ${PO}_{4}^{3-}$ was removed to various degrees in A0, B0, and C0 (Figure 4A). The concentration of ${PO}_{4}^{3-}$ decreased by 62.1%, 88.0%, and 91.1% in A0, B0, and C0, and the corresponding $k_{obs}$ values were 0.28, 0.71, and 0.73 h^−1^, respectively. Specifically, the removal efficiencies of ${PO}_{4}^{3-}$ in B0 and C0 were close, and B0 and C0 could be considered to possess similar MAP precipitation effects within 3 h. These results revealed that the MAP precipitation rate could be increased with a certain increase in bacterial solution concentration. Additionally, the slopes of the ${PO}_{4}^{3-}$ removal curves in B0 and C0 were almost level at 3 h, suggesting that the reaction was close to the termination point. This phenomenon could be mainly attributed to the limiting reactant concentrations (i.e., ${Mg}^{2+}$ ; ${PO}_{4}^{3-}$ ,; ${NH}_{4}^{+}$ ), and the MAP concentration would not increase with increasing bacteria concentration (Eq. 4).

Figures 4C, D shows the elimination of ${PO}_{4}^{3-}$ and the corresponding $k_{obs}$ values. The results showed that ${PO}_{4}^{3-}$ consumption of A1, B1, and C1 was less than 10% at 3 h. In order to further explore the consumption of ${PO}_{4}^{3-}$ by bacterial activity, the consumption of ${PO}_{4}^{3-}$ within 24 h was recorded. At 24 h, the ${PO}_{4}^{3-}$ consumption of A1 and B1 was less than 10%, and C1 was less than 20%. They were still far less than A0, B0, and C0. Therefore, it could be considered that ${PO}_{4}^{3-}$ -P consumption of cell life activity is negligible.

The medium also had the ability to precipitate MAP due to the presence of ${NH}_{4}^{+}$ . As shown in Figure 4E, ${PO}_{4}^{3-}$ was gradually eliminated with the progression of the reaction, and precipitation was observed in A2, B2, and C2 within 3 h. The ${PO}_{4}^{3-}$ removal rate in A2, B2, and C2 reached 24.4%, 33.3%, and 43.4%, respectively, within 3 h, while the mineralization effects in these three groups were similar to those in A0, B0, and C0. Therefore, the precipitation effect was also significantly improved with increasing culture medium concentration. This phenomenon could be mainly ascribed to the increase in ${NH}_{4}^{+}$ concentration in the environment with increasing culture medium concentration; the mineralization rate of MAP was further improved.

In addition, in the presence of microorganisms, the mineralization rate of MAP and the removal of ${PO}_{4}^{3-}$ were significantly accelerated. As displayed in Figures 4B, F, compared with A0, B0, and C0, the $k_{obs}$ value decreased in A2, B2, and C2 (culture medium absence of microorganisms). Within 3 h, the elimination rates of ${PO}_{4}^{3-}$ in A1 and B1 were 60.7% and 62.2% higher than those in A2 and B2, respectively. This result suggested that the microbial surface could serve as the nucleation site for precipitate mineralization, and the precipitation rate of MAP could be further accelerated. In addition, the rate of decrease in ${PO}_{4}^{3-}$ concentration in C0 was 52.3% higher than that in C2 at 3 h (Figures 4A, E). This phenomenon further revealed that due to the limiting reactant concentrations (i.e., ${Mg}^{2+}$ ; ${PO}_{4}^{3-}$ ; ${NH}_{4}^{+}$ ), the MAP concentration would not increase with increasing bacterial concentration. In summary, although both *S. pasteurii* and its culture medium had the ability to precipitate MAP, *S. pasteurii* played the major role in the mineralization process.

#### 3.1.2 Indirect ammonium source

**FIGURE 5:**
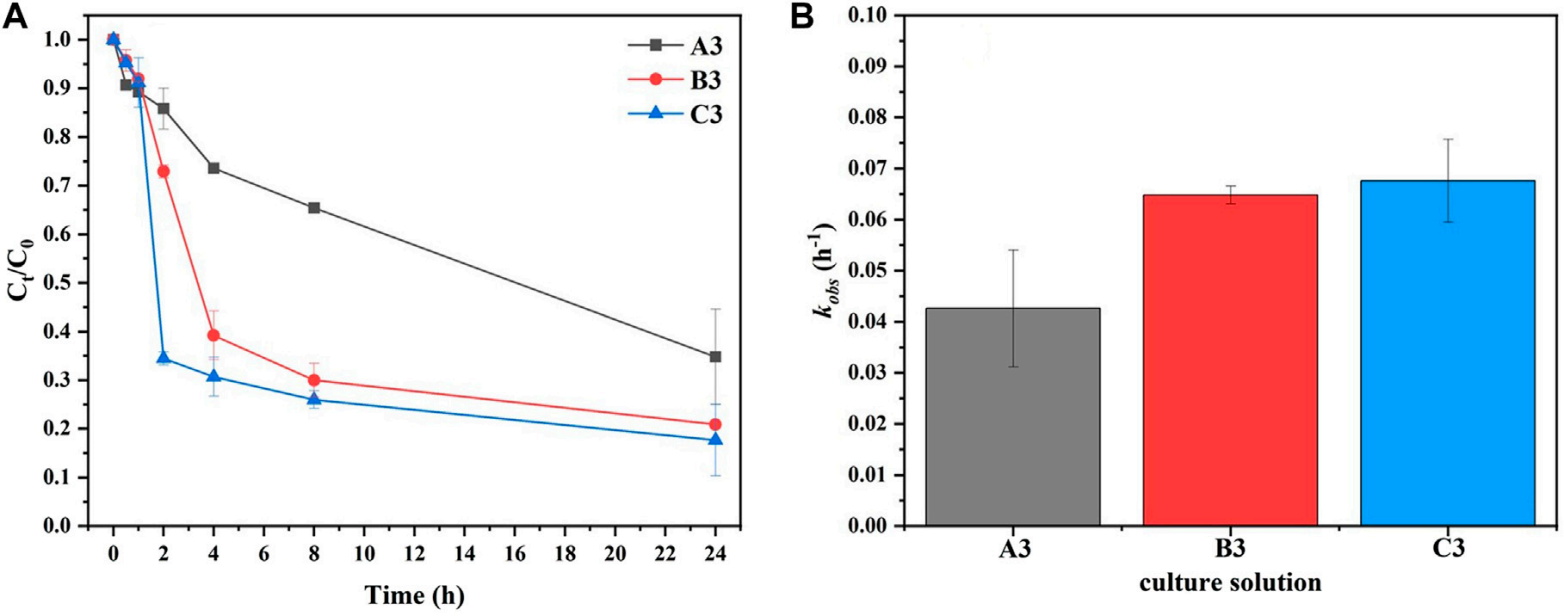
**(A)** The elimination of ${PO}_{4}^{3-}$ and **(B)** the corresponding *k*_*obs*_ values with an indirect ammonium source in MISP.

Figure 5 shows the effect of various bacterial solution concentrations (A3, B3, and C3) on ${PO}_{4}^{3-}$ removal and the corresponding $k_{obs}$ values with the addition of an indirect ammonium source. As shown in Figure 5, there was significant elimination of ${PO}_{4}^{3-}$ in A3, B3, and C3. Specifically, the removal efficiencies of ${PO}_{4}^{3-}$ in A3, B3, and C3 were 65.2%, 79.1%, and 82.3% within 24 h, and the corresponding $k_{obs}$ values were 0.043, 0.065, and 0.068 h^−1^, respectively. Therefore, considering that B3 and C3 exhibited similar MAP precipitation effects within 24 h, a bacterial concentration of ${OD}_{600}=0.181$ was adopted in subsequent experiments.

Interestingly, the concentrations of ${PO}_{4}^{3-}$ in A3, B3, and C3 decreased by 14.2%, 27.1%, and 65.5%, respectively, within 2 h. Since bacteria contain a certain amount of ${NH}_{4}^{+}$ and the concentration of bacteria was high in C3, the removal rate of ${NH}_{4}^{+}$ in C3 was much lower than that in A3 and B3. Thus, the rapid decrease in ${PO}_{4}^{3-}$ concentration within 2 h in C3 could be attributed to the amounts of ${NH}_{4}^{+}$ added along with the bacteria in C3 instead of produced by bacterial hydrolysis of urea. These results further revealed that the amounts of ${NH}_{4}^{+}$ contained in the bacteria of A3 and B3 were insufficient to achieve the MAP precipitation reaction. Additionally, the slopes of the ${PO}_{4}^{3-}$ elimination curve in B3 and C3 were almost level after 4 h, suggesting that urea was hydrolyzed by the metabolic action of bacteria in the system and then ${NH}_{4}^{+}$ was released for MAP mineralization.

Previous studies reported that bacterially induced MAP crystallization took more than 72 h to consume most of the ${PO}_{4}^{3-}$ in the system (Sinha et al., 2014; Zhao et al., 2019). Interestingly, the concentration of ${PO}_{4}^{3-}$ was reduced by 79.1% within 24 h in this study. In the experiment, the reactants were added to the culture medium of bacteria to be resuscitated, and the process of mineralization included the process of bacterial recovery and growth. Therefore, mineralization could be effectively accelerated with the large-scale cultivation of sufficient amounts of bacteria in solution during the experiment.

### 3.2 Optimization of the MISP mineralization scheme

**FIGURE 6:**
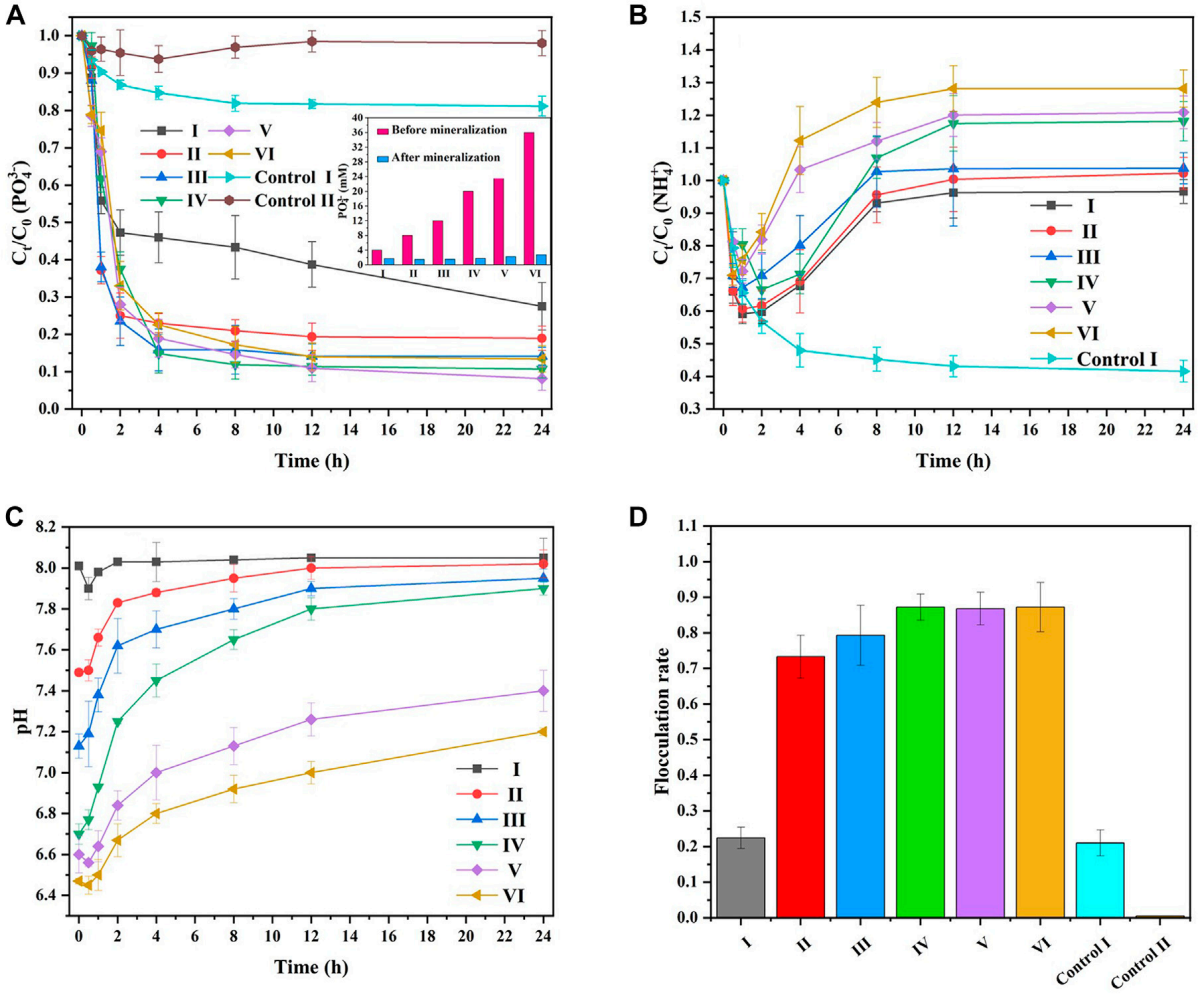
The elimination of **(A)** ${PO}_{4}^{3-}$ (inset represents the concentration of ${PO}_{4}^{3-}$ corresponding to the experimental group), **(B)** ${NH}_{4}^{+}$ , **(C)** the change in pH, and **(D)** the change in flocculation rate with MISP.

Figure 6 shows the effect of different groups (Table 2) on ${PO}_{4}^{3-}$ and ${NH}_{4}^{+}$ elimination, pH, and flocculation rate. As displayed in Figure 6A, the decrease rate in ${PO}_{4}^{3-}$ concentration from Group II to Group VI was rapid from 0 to 4 h and then slowed with further progression of the reaction from 4 to 24 h, similar to the phenomenon in Section 3.1.2.

The concentration of ${PO}_{4}^{3-}$ in Control I also decreased, owing to the presence of ${NH}_{4}^{+}$ in the medium (similar to A2, B2, and C2), while the ${PO}_{4}^{3-}$ concentration did not decrease in Control II, owing to the absence of ${NH}_{4}^{+}$ for MAP mineralization. Moreover, the removal efficiency of ${PO}_{4}^{3-}$ in Group I was much lower than that in the other experimental groups, and the elimination rate improved from Group I to Group VI. However, the inset of Figure 6A shows that the concentrations of ${PO}_{4}^{3-}$ in Group I to Group IV were almost the same at the end of the reaction but the concentrations in Groups V and VI were slightly higher. These results could be attributed to the fact that the initial concentration of ${PO}_{4}^{3-}$ was different from Group I to Group Ⅵ, and the dynamic equilibrium existed between the concentration of ${PO}_{4}^{3-}$ and the yield of MAP at 24 h. Therefore, the bacterial concentration of ${OD}_{600}=0.181$ exhibited a urease surplus with low reactant concentrations. In all the experimental groups, a small amount of ${PO}_{4}^{3-}$ remained in the liquid phase after the reaction. This result could be ascribed to the reversible reaction in Eq. 4; dynamic equilibrium existed between ions and MAP in the solution. In addition, with the precipitation of MAP, the pH of the system decreased, suggesting incomplete precipitation.

Generally, the decrease in ${PO}_{4}^{3-}$ concentration was accompanied by the consumption of ${NH}_{4}^{+}$ . As seen, ${NH}_{4}^{+}$ was gradually consumed in Control I (Figure 6B). In contrast to ${PO}_{4}^{3-}$ , ${NH}_{4}^{+}$ was released by the metabolic activities of bacteria, so the concentration of ${NH}_{4}^{+}$ in all the experimental groups showed an increase from 2 h to 8 h after rapid removal. The concentration of ${PO}_{4}^{3-}$ also decreased with the removal of ${NH}_{4}^{+}$ within 4 h (Figure 6A). This result could be interpreted by the combined effect of the ${NH}_{4}^{+}$ produced by urea hydrolysis by bacteria with the reaction of ${NH}_{4}^{+}$ and ${PO}_{4}^{3-}$ in the system at nucleation sites. This combined effect caused the rate of decrease in ${PO}_{4}^{3-}$ concentration to be much higher than the rate of ${NH}_{4}^{+}$ released from microbial metabolism and further confirmed that the release of ammonia could be reduced in the process of mineralization with MISP technology. The concentration of ${NH}_{4}^{+}$ increased and reached approximately 1.2 times the initial concentration from 2 h to 8 h (Figure 6B), while the concentration of ${PO}_{4}^{3-}$ slightly decreased, the removal rate reached 80.0%–90.0%, and the slope of the removal curve was stable (Figure 6A). These phenomena could be attributed to the ${PO}_{4}^{3-}$ concentration at 2 h–8 h not being sufficient to rapidly drive the reaction to the right in Eq. 4. ${NH}_{4}^{+}$ released by microbial metabolic activities accumulated in the system, resulting in an increase in ${NH}_{4}^{+}$ concentration. The rate of decrease in ${PO}_{4}^{3-}$ concentration was lower than that of microbial hydrolysis of urea. From 8 to 24 h, the concentration of ${NH}_{4}^{+}$ nitrogen remained level, indicating that urea was completely hydrolyzed. However, from Group I to Group Ⅵ, the concentration of ${NH}_{4}^{+}$ nitrogen gradually increased. This is because as the concentration of the reactants (i.e., PBS, urea) increased, the concentration of the components (i.e., ${PO}_{4}^{3-}$ ; ${NH}_{4}^{+}$ ) as they reached dynamic equilibrium also increased. This is consistent with the results shown in the inset of Figure 6A.

Hydrolysis of urea can increase surrounding pH, and the formation of struvite can reduce surrounding pH. As shown in Figure 6C, the pH of all groups decreased at 0–0.5 h. And then increased and approached pH 8 after 0.5 h. This is because ${NH}_{4}^{+}$ in the bacterial solution was mineralized into MAP, and the precipitation rate of MAP is higher than the hydrolysis rate of urea. With the consumption of Mg^2+^ concentration and the increase of ${NH}_{4}^{+}$ concentration, pH began to rise after 0.5 h. In addition, the higher the concentration of the reactant, the lower the pH at the beginning of the reaction. This is because MgCl_2_ is acidic. And the reaction solution pH could not be well buffered by PBS.

As shown in Figure 6D, from Group I to Group IV, the flocculation rates were 22.4%, 73.3%, 85.3%, and 87.2%, respectively, suggesting that the flocculation rate improved with increasing reactant concentration. Group I exhibited a 22.4% flocculation rate, and Control I exhibited a 21.0% flocculation rate, indicating that the flocculation effect could also be observed in the presence of the culture medium. This result revealed that MAP could be mineralized and precipitated between kaolin particles in the MISP system. Therefore, there existed a synergistic effect between the decrease in ${PO}_{4}^{3-}$ concentration and flocculation in the MISP system.

In summary, 10 mM urea, 20 mM PBS, and 25 mM ${MgCl}_{2}$ should be used to achieve the best flocculation effect.

### 3.3 Effect of initial pH

**FIGURE 7:**
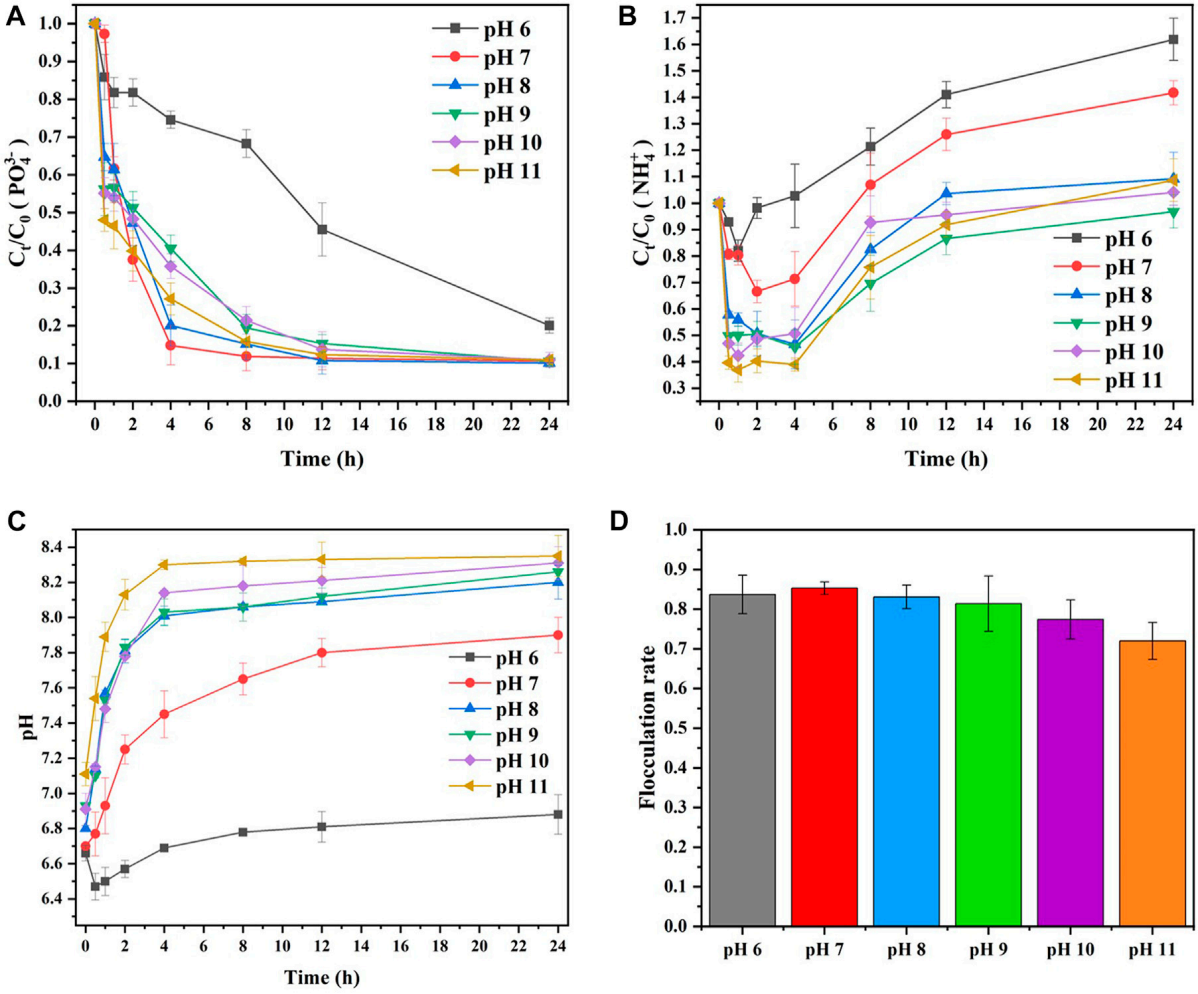
Effect of initial pH on the elimination of **(A)** ${PO}_{4}^{3-}$ and **(B)** ${NH}_{4}^{+}$ , **(C)** the change in pH, and **(D)** the change in flocculation rate with MISP.

Generally, pH 9 is the optimum condition for MAP mineralization and precipitation with non-microbial processes (Jaffer et al., 2002), while MAP precipitation induced by bacteria has an excellent effect at pH values between 7.3 and 8.3 (Tansel et al., 2018). Therefore, it is necessary to explore the influence of pH on MAP mineralization and precipitation in MISP technology. Figure 7 shows the effect of pH on the ${PO}_{4}^{3-}$ and ${NH}_{4}^{+}$ elimination, the pH, and the flocculation rate in the MISP system.

As displayed in Figure 7A, the elimination efficiency of ${PO}_{4}^{3-}$ within 24 h reached 79.9% under pH 6 and 98.5% under other pH conditions (i.e., 7, 8, 9, 10, and 11), indicating that acidic conditions were unfavorable for MAP mineralization and precipitation with the MISP system. Interestingly, white floccules precipitated at the beginning of the reaction at pH 10 and 11, suggesting that the amorphous magnesium phosphate or magnesium hydroxide precipitation could be generated with the addition of reactants (i.e., ${Mg}^{2+}$ ; ${PO}_{4}^{3-}$ ; ${NH}_{4}^{+}$ ) under strongly alkaline conditions.

The ${NH}_{4}^{+}$ concentration could reflect the ability of bacteria to hydrolyze urea. Figure 7B showed the effect of pH on ${NH}_{4}^{+}$ removal in the MISP system. As can be seen, ${NH}_{4}^{+}$ concentration decreased within 2 h and then had a gradual increase with the processing of the reaction under pH 6 to 11 (Figure 7B). Moreover, the accumulated efficiency of ${NH}_{4}^{+}$ concentration could be improved with the decrease of the reaction pH, indicating that the acidic PBS conditions were not conducive to the removal of ${PO}_{4}^{3-}$ (Figure 7A) and further inhibited the process of MAP mineralization in the MISP system.

As shown in Figure 7C, the reaction solution pH could not be well buffered by PBS because of the added acidic MgCl_2_. The initial pH of the reaction is between 6.6 and 7.1. Then, the pH increased gradually as the reaction progressed. It was because the ${NH}_{4}^{+}$ concentration was increased by microbial metabolic activities (Figure 7B).

${PO}_{4}^{3-}$ forms an amorphous precipitate and has a negative effect on flocculation with increasing pH (Moulessehoul et al., 2017). Figure 7D shows the effect of pH on the flocculation rate in the MISP system. The flocculation rate decreased with the increase of pH from 7 to 11, suggesting that the elimination of ${PO}_{4}^{3-}$ and ${Mg}^{2+}$ was accelerated with increasing reaction pH. Thus, the production of MAP was reduced, the cohesion factor for kaolin in the system was decreased, and the flocculation process was further inhibited. In addition, the flocculation rate at pH 6 was slightly lower than that at pH 7. This result revealed that acidic pH conditions were not conducive to the generation of MAP, resulting in a decrease in cohesion factors for kaolin in the system and a further decline in the flocculation rate.

Therefore, the flocculation rate was related to the utilization rate of ${PO}_{4}^{3-}$ for MAP formation from the reactants, and the MISP system had the best flocculation efficiency at pH 7.

### 3.4 Effect of $Cu^{2+}$

The waste mud contained a certain concentration of ${Cu}^{2+}$ , and ${Cu}^{2+}$ can adsorb onto MAP crystals in the liquid phase (Peng et al., 2018). Therefore, the influence of ${Cu}^{2+}$ on MISP-driven mud treatment was investigated.

**FIGURE 8:**
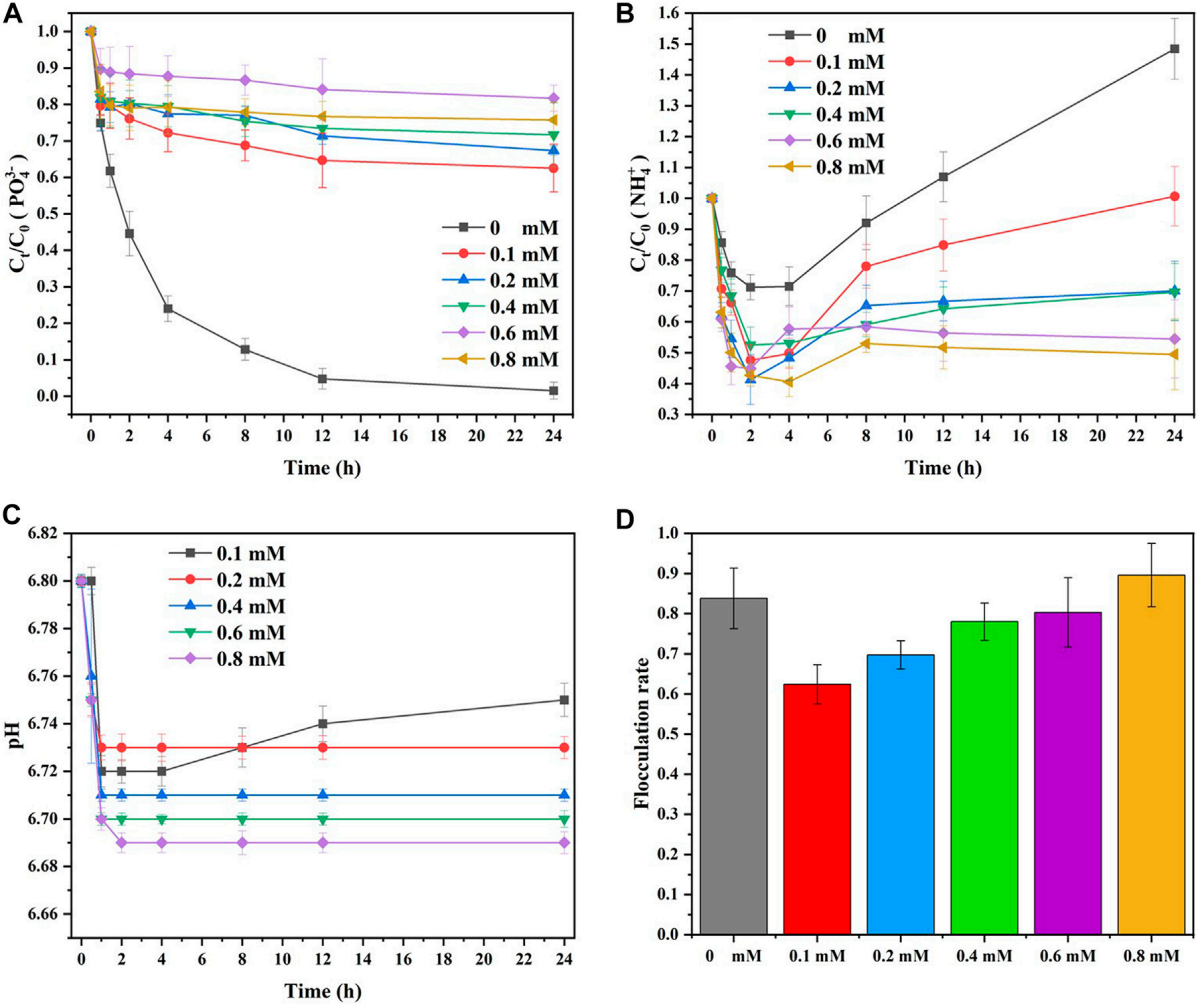
Effect of Cu^2+^ concentration on the elimination of **(A)** ${PO}_{4}^{3-}$ and **(B)** ${NH}_{4}^{+}$ , **(C)** the change in pH, and **(D)** the change in flocculation rate with MISP.

Figure 8 shows the effect of ${Cu}^{2+}$ concentration on ${PO}_{4}^{3-}$ and ${NH}_{4}^{+}$ elimination and the flocculation rate in the MISP system. As shown in Figure 8A, the elimination efficiency of ${PO}_{4}^{3-}$ was 98.5% after 24 h without the addition of ${Cu}^{2+}$ in the MISP system. The removal rate of ${PO}_{4}^{3-}$ significantly decreased to 37.5%, 32.7%, 28.4%, 18.3%, and 24.3%, respectively, with increasing addition of ${Cu}^{2+}$ (0.1, 0.2, 0.4, 0.6, and 0.8 mM) to the MISP system. Interestingly, the elimination of ${PO}_{4}^{3-}$ markedly accelerated within 1 h with the addition of 0.6 and 0.8 mM ${Cu}^{2+}$ , and the removal rate of ${PO}_{4}^{3-}$ was higher with 0.8 mM ${Cu}^{2+}$ than with 0.6 mM ${Cu}^{2+}$ . A previous study reported that with the addition of a high concentration of ${Cu}^{2+}$ ; ${Cu}^{2+}$ will be combined with ${PO}_{4}^{3-}$ , and then amorphous copper phosphate precipitates will be generated (Lu et al., 2021). Therefore, the sudden decrease in ${PO}_{4}^{3-}$ concentration within 1 h and the higher removal rate of ${PO}_{4}^{3-}$ with 0.8 mM ${Cu}^{2+}$ could be attributed to the combination of ${Cu}^{2+}$ and ${PO}_{4}^{3-}$ .

As shown in Figure 8B, the concentration of ${NH}_{4}^{+}$ decreased within 4 h and then increased to various degrees. Specifically, with increasing addition of ${Cu}^{2+}$ , the accumulation of ${NH}_{4}^{+}$ significantly slowed from 4 to 24 h. This phenomenon could be attributed to the addition of ${Cu}^{2+}$ having a negative effect on the metabolic activity of bacteria. Since ${PO}_{4}^{3-}$ decreased slowly and ${NH}_{4}^{+}$ was also consumed, the rate of bacterial hydrolysis of urea could be considered to be less than the rate of MAP mineralization with the addition of more than 0.4 mM ${Cu}^{2+}$ . Therefore, ${Cu}^{2+}$ had a strongly negative effect on the removal of ${PO}_{4}^{3-}$ and further inhibited the mineralization of MAP.

As shown in Figure 8C, the reaction solution pH was not alkaline because the added solutions of MgCl_2_ and CuCl_2_ were acid and could not be well buffered by PBS. Mg^2+^ and Cu^2+^ could not coexist with ${PO}_{4}^{3-}$ and would react with ${PO}_{4}^{3-}$ to transform into the amorphous precipitates [i.e., Mg_3_(PO_4_)_2_ and Cu_3_(PO_4_)_2_]. The amorphous precipitates would not be generated when the reaction solution reached the ionization equilibrium. 

Owing to the inhibition effect of the added ${Cu}^{2+}$ , bacterial activity had a negative effect on flocculation. Figure 8D shows the influence of ${Cu}^{2+}$ concentration on the flocculation rate. The flocculation rate with the addition of 0.1 mM ${Cu}^{2+}$ in the MISP system was approximately 20% lower than that without ${Cu}^{2+}$ , suggesting that added ${Cu}^{2+}$ could affect the metabolic activity of bacteria and further inhibit the mineralization rate of MAP. Moreover, since there were insufficient MAP crystals in the MISP system for kaolin particles to attach, the flocculation rate significantly decreased. However, the flocculation rate increased with increasing ${Cu}^{2+}$ concentration (Figure 8D). Specifically, the flocculation rate with 0.8 mM ${Cu}^{2+}$ in the MISP system was even higher than that without ${Cu}^{2+}$ in the solution. ${Cu}^{2+}$ is a heavy metal cation, and the edges of kaolin particles are negatively charged (Mouni et al., 2018). Consequently, negatively charged kaolin particles were attracted to ${Cu}^{2+}$ and then formed larger particles. ${Cu}^{2+}$ was trapped in the intergranular cavities (Chaouf et al., 2019) and more easily settled under gravity. Therefore, the flocculation induced by ${Cu}^{2+}$ was enhanced with increasing ${Cu}^{2+}$ concentration. Many researchers have used this principle of adding anionic flocculants to liquid phases containing heavy metals, such as ${Cu}^{2+}$ (Wu et al., 2018; Chaouf et al., 2019), to remove heavy metals. 

In summary, ${Cu}^{2+}$ had a significant inhibitory effect on the urease activity of bacteria, and the inhibition of urease activity was significant with increasing ${Cu}^{2+}$ concentration. However, since negatively charged kaolin particles were attracted to heavy metal cations and then formed larger particles that settled under gravity, flocculation was enhanced with increasing ${Cu}^{2+}$ concentration. Therefore, the effect of heavy metal ions played an important role in flocculation and should be considered in the application of the MISP technology.

### 3.5 Characteristics of precipitates

To verify their specific composition, the precipitates were collected, washed, and dried according to the method described in Section 2.4.4, and then the material composition, morphology, and elemental composition were analyzed by XRD, SEM, and TEM-EDS.

**FIGURE 9:**
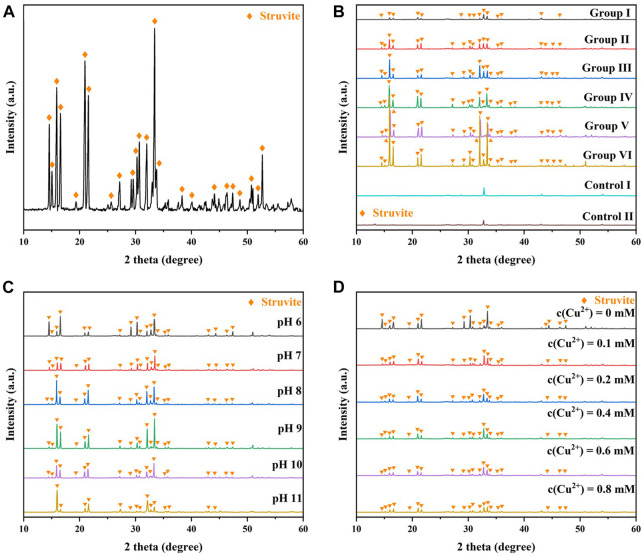
**(A)** XRD patterns of MAP and XRD patterns of precipitates for **(B)** different groups, **(C)** different pH values, and **(D)** different concentrations of ${Cu}^{2+}$ .

As shown in Figure 9A, the XRD patterns of the precipitates of B3 showed strong typical MAP diffraction peaks at 16°, 21°, and 33° (Thamaphat et al., 2008), suggesting that MAP was the main component of the precipitates. Figure 9B shows the XRD patterns of the different groups in Section 3.2. The kaolin from Control I and Control II showed strong diffraction peaks at 29°, 33°, and 43°, while the patterns at other angles were flat. The XRD patterns of the flocculated products in the experimental group showed that in addition to the original diffraction peaks for kaolin, stronger MAP diffraction peaks at 16°, 21°, and 33° could be detected. These results further indicated that MAP was the main component of the flocculation products obtained from the above experiments. With increasing reactant concentration, the diffraction peaks of the XRD patterns at 16°, 21°, and 33° became stronger (Figure 9B), suggesting that the amount of MAP in the mineralized products accumulated with increasing reactant concentration. Interestingly, from Group I to Group III, XRD peaks could also be detected at angles near 16°, 21°, and 33°, and the diffraction peaks became stronger with increasing reactant concentration. However, from Group IV to Group VI, diffraction peaks only appeared at 16°, 21°, and 33° and were stronger than those in Group I to Group III. These results indicated that increasing the concentration of the reactants was conducive to the generation of MAP crystals with a single shape in the flocculated products.

Figure 9C shows the effect of initial pH on the XRD patterns of the reaction precipitates. MAP crystallites are more disorganized in the initial pH 6. The initial pH from 7 to 9, the higher the initial pH, the more monotonous the crystal of the mineralized MAP. Figure 9D shows the effect of ${Cu}^{2+}$ concentration on the XRD patterns of the reaction precipitates. All the patterns showed strong diffraction peaks at 15°, 21°, and 33°, while the diffraction peaks gradually weakened with increasing ${Cu}^{2+}$ concentration. This phenomenon could be interpreted as follows: ${Cu}^{2+}$ interfered with the growth of struvite crystals, promoted the formation of the amorphous phase, and further inhibited the precipitation of MAP (Lu et al., 2021).

**FIGURE 10:**
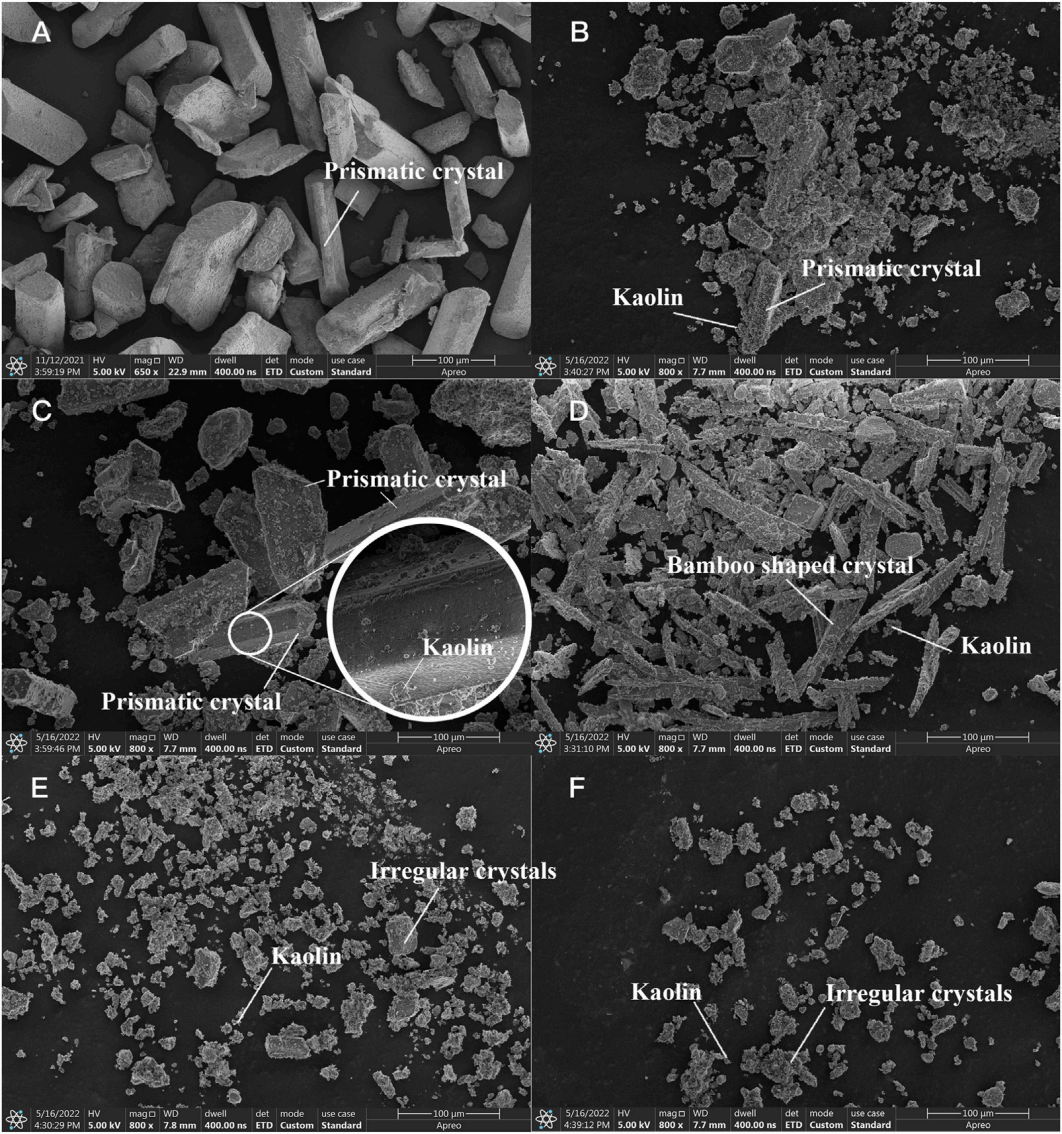
SEM images of precipitates from **(A)** B3, **(B)** Group I, **(C)** Group IV, **(D)** pH 11, **(E)** $c\left({Cu}^{2+}\right)=0.1\;mM$ , and **(F)** $c\left({Cu}^{2+}\right)=0.2\;mM$ .

MAP obtained from different methods or microorganisms exhibits different crystal shapes, usually needle-like (Chauhan and Joshi, 2013) or rod-like shapes (Stolzenburg et al., 2015). Therefore, SEM was carried out to detect the MAP morphology. Figure 10A shows SEM images of the MAP precipitates in B3 under the microbial mineralization described in Section 3.1.2. The MAP crystals had a length of 60–130 μ m, prism shapes tilted at both ends, and a trapezoidal longitudinal section.

Figure 10B show SEM photos of the flocculation products in Group I and Group IV. Although similar to the crystal shape shown in Figure 10A, the crystals observed in Group I and Group IV contained some extra matter on the surface (Figure 10B). This result indicated that the kaolin particles suspended in the MISP system can also serve as nucleation sites for MAP crystals, settle under gravity, and finally achieve flocculation. The mineralized crystals were small in volume and mass, prismatic in shape, and different in type (Figure 10B) because of the low concentration of reactants in Group I. As the reactant (i.e., ${Mg}^{2+}$ ; ${PO}_{4}^{3-}$ ; ${NH}_{4}^{+}$ ) concentration increased, the volume of the crystals increased (Figure 10B). Most of the crystals were prismatic particles with inclined ends (Figure 10C), consistent with the XRD patterns shown in Figure 9B.

According to a previous study, the shape of crystals changes under strongly alkaline pH conditions (Prywer et al., 2012). Figure 10D shows SEM imagines of the flocculated products at pH 11. The crystals were in the form of elongated rods with floccules attached to the crystal surface under strongly alkaline pH conditions. However, this was not a normal crystal shape for MAP and might have been caused by the high pH.

Figures 10E,F show SEM images of precipitates formed with 0.1 and 0.2 mM ${Cu}^{2+}$ . Small and irregular MAP crystals were found in the precipitates with the addition of ${Cu}^{2+}$ in the MISP system (Figure 10E) and were five to seven times shorter than those detected without the addition of ${Cu}^{2+}$ (Figure 10C). With increasing ${Cu}^{2+}$ concentration, the number of small MAP crystals decreased (Figures 10E,F), consistent with the phenomenon shown in Figure 9D.

**FIGURE 11:**
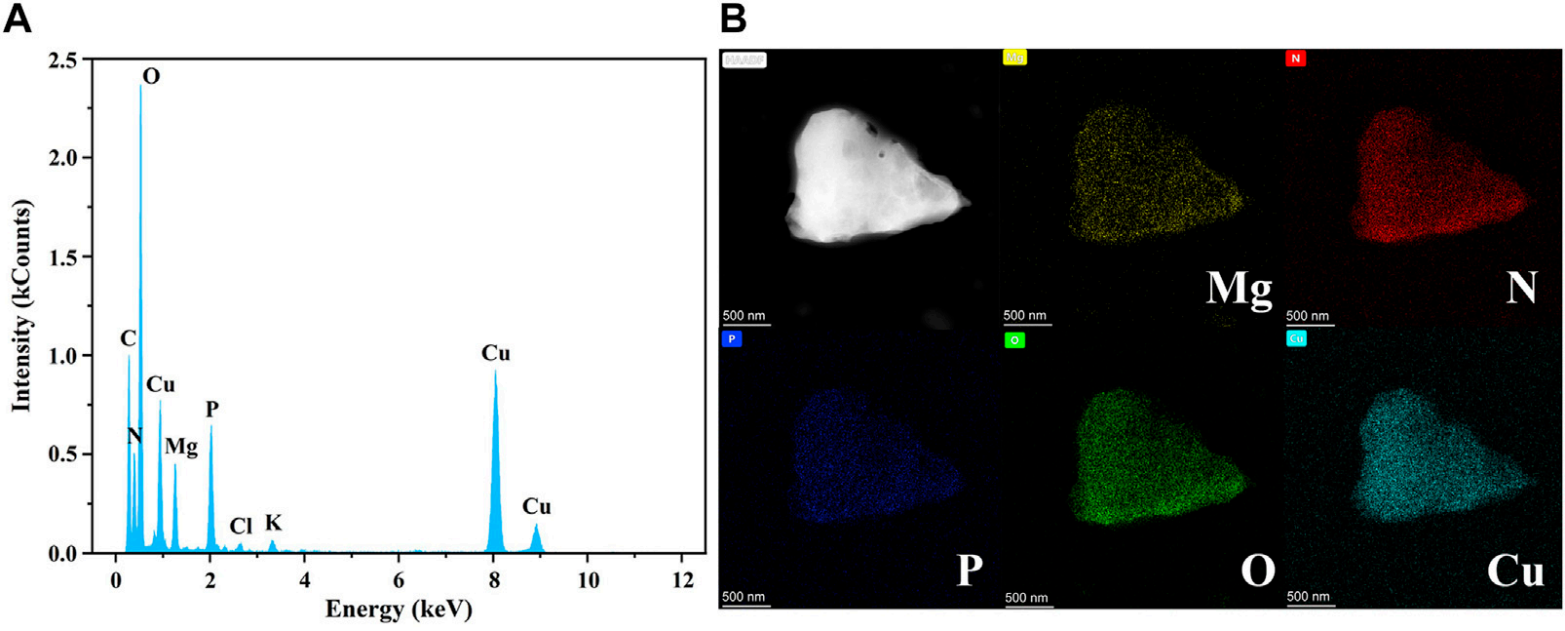
**(A)** EDS analysis and **(B)** TEM elemental mapping images of precipitates from $c\left({Cu}^{2+}\right)=0.8\;mM$ .

Figure 11 shows the EDS analysis results and TEM elemental mapping images of precipitates formed with 0.8 mM ${Cu}^{2+}$ . In addition to Mg, N, P, and O, C was found in the EDS analysis (Figure 11A). C is the basic element of life and is ubiquitous in bacterial cells. The presence of C suggested that bacteria served as the nucleation sites. As the elements that makeup MAP, Mg, N, P, and O elements had roughly the same distributions in the EDS results (Figure 11B). This result was consistent with the XRD patterns (Figure 9D). In addition, P and O were more densely distributed than Mg, and Cu was also discovered in the EDS analysis and TEM images. This shows that Cu was combined with P and O and fixed in the precipitate. This confirmed that amorphous copper phosphate precipitates were generated with the addition of a high concentration of ${Cu}^{2+}$ , as mentioned in Section 3.4.

**FIGURE 12:**
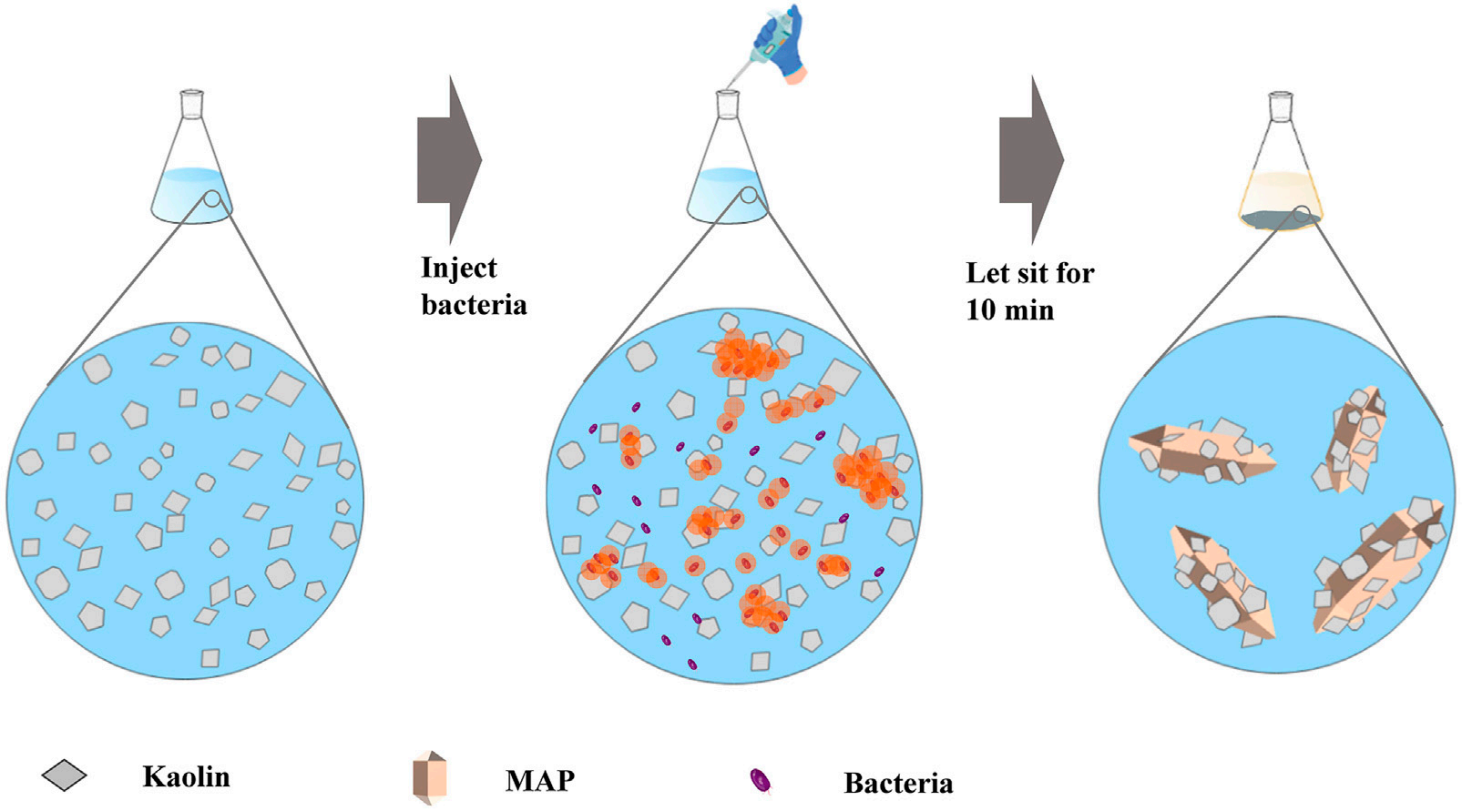
Mechanism of MISP for treating kaolin.

Therefore, with suitable mineralization conditions, ${NH}_{4}^{+}$ and ${CO}_{3}^{2-}$ were produced by urea decomposition under bacterial metabolic activities, and the generated ${NH}_{4}^{+}$ further combined with ${PO}_{4}^{3-}$ and ${Mg}^{2+}$ in the solution on the bacterial surface to form MAP. Kaolin particles acted as nucleation sites to bind the generated MAP crystals and then settled under gravity (Figure 12).

### 3.6 Costs

The costs of common inorganic flocculants and organic flocculants were compared to assess the cost of the MISP mud treatment. Different flocculants need different dosages to achieve the best flocculation efficiency. A previous study reported that mixing different inorganic flocculants with 20 mL acrylamide solution (52%) could achieve the best flocculation efficiency for mud treatment. ${FeCl}_{3}$ or ${Al}_{2}{\left({SO}_{4}\right)}_{3}$ contributes 1,286 mg/g dry matter, and polyaluminium chloride (PAC) or polyferric chloride (PFC) contributes 1,000 mg/g dry matter (Qi et al., 2022). The unit price of acrylamide is CNY 19.8 per kg, and CNY 17.17 per kg of dry matter will be used to treat waste mud.

The cost of the MISP technology mainly consists of the costs of the culture medium and ${MgCl}_{2}$ . Ammonium and phosphorus can be obtained from polluted water bodies, municipal sludge ash, and aquaculture wastewater (Sathiasivan et al., 2021; Thant Zin and Kim, 2021). Bacteria could be grown in the prepared medium, and a small number of strains were cultured for 48 h. Therefore, the MISP technology further reduced the cost of industrial mud treatment. Table 3 shows the cost of different types of flocculants in terms of their current market value and the unit price to achieve the same amount of dry matter flocculation. Compared with the combined treatments of organic and inorganic flocculants or inorganic polymer flocculants, the MISP technology could remarkably reduce costs. In addition, organic flocculants, such as acrylamide, can cause secondary pollution (Faouzi et al., 2018).

**TABLE 3:** Cost at current market value^a^ and dosage of flocculant.

Method	Materials	Concentration (kg/L)	Unit price (CNY/kg)	Dosage (L (kg)/kg dry matter)	Price (CNY/kg dry matter)
Acrylamide + ${FeCl}_{3}$	Acrylamide	0.52000	19.80	0.87	21.30
	${FeCl}_{3}$	-	3.20	1.29	
Acrylamide + ${Al}_{2}{\left({SO}_{4}\right)}_{3}$	Acrylamide	0.52000	19.80	0.87	21.30
	${Al}_{2}{\left({SO}_{4}\right)}_{3}$	-	3.20	1.29	
Acrylamide + PAC	Acrylamide	0.52000	19.80	0.87	20.77
	PAC	-	3.60	1.00	
Acrylamide + PFC	Acrylamide	0.52000	19.80	0.87	20.77
	PFC	-	3.60	1.00	
MISP	${MgCl}_{2}$	0.00508	5.80	1.00	4.19
	YE	0.02000	32.00		
	${\left({NH}_{4}\right)}_{2}{SO}_{4}$	0.01000	3.20		
	Trisaminomethane	0.01575	258.00		

^a^ The unit price was obtained from Alibaba.com.

In summary, the MISP technology possesses a relatively low environmental impact and costs, and the process would have good applications in the treatment of engineering waste mud.

## 4 Conclusion

In this study, MISP technology was introduced into the field of mud treatment to induce solid settling. The mechanism of MISP technology for mud treatment was explored, and the scheme for mineralization during the treatment of mud was optimized. The main conclusions are as follows:

(1) The flocculation rate could reach about 87.2% under the optimum condition of mud treatment with MISP technology ( $8.36\times 10^{6}\;cell∙{ml}^{-1}$ bacteria, 10 mM urea, 20 mM PBS (pH 7), and 25 mM ${MgCl}_{2}$ ).
(2) XRD and SEM analysis showed that the MAP crystals, with a length of 60–130 μm, were prismatic and surrounded by kaolin particles under the optimum mineralization parameters.
(3) MISP technology could improve the fertility of the flocculated product and should prioritize the removal of heavy metals for soil applications.
(4) MISP technology was more advantageous in cost and environmentally friendly than other methods to treat waste mud.
(5) In the real application, the depth of the mud pool and the stillness of the pool water would be the challenges with MISP. The injection of air into the mud pool and the stirring of the mud pool could improve MISP technology to treat waste mud.

## Data Availability

The original contributions presented in the study are included in the article/supplementary material, further inquiries can be directed to the corresponding author.
